# Nutrition in critical illness: a current conundrum

**DOI:** 10.12688/f1000research.9278.1

**Published:** 2016-10-18

**Authors:** L. John Hoffer, Bruce R. Bistrian

**Affiliations:** 1 Lady Davis Institute for Medical Research, Jewish General Hospital, McGill University, Montreal, QC, Canada; 2 Beth Israel Deaconess Medical Center, Harvard Medical School, Boston, MA, USA

**Keywords:** ICU nutrition, crtically ill, catabolic, enteral nutrition, parenteral nutrition

## Abstract

Critically ill people are unable to eat. What’s the best way to feed them? Nutrition authorities have long recommended providing generous amounts of protein and calories to critically ill patients, either intravenously or through feeding tubes, in order to counteract the catabolic state associated with this condition. In practice, however, patients in modern intensive care units are substantially underfed. Several large randomized clinical trials were recently carried out to determine the clinical implications of this situation. Contradicting decades of physiological, clinical, and observational data, the results of these trials have been claimed to justify the current practice of systematic underfeeding in the intensive care unit. This article explains and suggests how to resolve this conundrum.

## Introduction

Critical illness is defined in various ways. It is commonly regarded as any disease that requires treatment in an intensive care unit (ICU). This article focuses on a particular form of critical illness which we term “catabolic critical illness.” Catabolic critical illness is the life-threatening condition created by overwhelming infection, trauma, or other kinds of severe tissue injury. The defining characteristic of catabolic critical illness is the body’s inflammatory response to major injury, a coordinated cytokine-, hormone-, and nervous system-mediated phenomenon that alters temperature regulation and energy expenditure, invokes neuroendocrine and hematologic responses, changes the synthesis and disposition of certain proteins in the body, and – of greatest importance for this article – dramatically stimulates muscle protein catabolism ^1– 3^. The protein-catabolic response to traumatic injury was described more than 80 years ago ^4, 5^; we now know that trauma is only one cause of catabolic critical illness. A common form of catabolic critical illness, sepsis, is the systemic inflammatory response that occurs with severe infection ^6^. More specifically, this article explains and analyzes a current controversy regarding the role of nutritional interventions to improve the clinical outcomes of patients with catabolic critical illness.

Critically ill patients are treated in ICUs that deploy one-on-one nursing and sophisticated life-support technologies. Before the modern era of sophisticated patient monitoring and care, surgery, pharmacology, and fluid management, most critically ill patients died. Nowadays, the overall in-hospital mortality of patients admitted to an ICU in the United States is approximately 12% ^7, 8^ and their average ICU stay is less than 4 days ^7^. These figures apply to patients admitted to an ICU for any reason, including for post-surgical monitoring or the management of gastrointestinal hemorrhage, cardiac arrest, primary respiratory, heart, or liver failure, and other diseases that are not necessarily associated with the severe tissue damage and systemic inflammatory response that characterize catabolic critical illness. In-hospital mortality and the length of an ICU stay are greater in Europe (approximately 23% and 12 days, respectively ^9^), presumably reflecting their different patient mix. In-hospital mortality from catabolic critical illness, especially sepsis, often reaches 25–50% ^7, 10^.

## Does nutrition have a role in critical illness?

Everyone needs adequate nutrition, but the argument for prompt nutritional interventions in catabolic critical illness is especially strong. The patient is too sick to eat, and even if food was offered it would be refused because the systemic inflammatory response creates a strong aversion to eating. The anorexic effect of systemic inflammation no doubt serves a biological function, but it is profoundly disadvantageous in prolonged systemic inflammation, which dramatically increases muscle protein catabolism and moderately increases energy expenditure. Unless mitigated by suitable specialized nutrient provision, the combination of dramatically increased body protein loss, increased calorie expenditure, and no food intake will precipitate severe muscle atrophy and diminish adipose tissue stores.

Catabolic critical illness increases energy expenditure, but it does so variably and for several reasons, many of which can be mitigated in modern ICUs (they include high fever, incessant restless movement, and loss of thermal insulation in patients with extensive skin burns). Moreover, most of the patients in modern ICUs have an ample supply of calories stored in their adipose tissue; a large proportion of them are frankly obese. One would presume, therefore, that short-term lack of calories is rarely an urgent problem in the ICU.

Protein, not calories, is the crucial macronutrient in catabolic critical illness. The body has only one store of protein, skeletal muscle, and catabolic critical illness rapidly exhausts it. The muscle atrophy of catabolic critical illness can be rapid and severe enough to debilitate healthy young adults whose initial muscle mass was normal. Yet many critically ill people (including ones who are obese) already suffer from generalized muscle atrophy when they are admitted to an ICU. The usual causes of muscle atrophy in these patients are old age, disuse muscle atrophy, and pre-existing protein-energy malnutrition (starvation disease) that made them vulnerable to the critical illness they have now developed.

## How does catabolic critical illness increase protein requirements?

Protein is an essential nutrient for adults because protein turnover involves a continuous breakdown (proteolysis) and recapture (protein synthesis) of endogenous amino acids that is less than perfectly efficient. Endogenous amino acid catabolism can be adaptively reduced in response to dietary protein deprivation, but not below a certain minimum known as the protein minimum or obligatory nitrogen (N) excretion rate (in this discussion, N and protein are used almost interchangeably. Proteins of nutritional interest are 16% N by weight. The excretion of 1 g N from the body – mostly as urea N – implies a loss from the body of 6.25 g formed protein). Obligatory N indicates the body’s maximum ability to recycle endogenous protein and its corresponding minimum rate of endogenous amino acid catabolism, so it is an important determinant of the minimum protein requirement. The minimum protein requirement is also affected by the efficiency with which the body incorporates exogenous (dietary) amino acids into body proteins; this process is also subject to adaptive regulation ^11^.

Obligatory protein loss and inefficient incorporation of exogenous amino acids into endogenous protein being unavoidable, a minimum amount of protein has to be consumed in the diet to make up for these losses. The currently accepted average minimum dietary protein requirement was identified by means of N balance experiments in which healthy volunteers were adapted to different diets varying in protein content. The lowest daily protein intake compatible with zero N balance – approximately 0.65 g/kg body weight – is the average normal minimum protein requirement. To account for inter-individual variability, an amount of protein equal to two standard deviations is added to this value to calculate the “safe” or “recommended” daily minimum protein intake of 0.80 g/kg normal body weight ^11^.

For many ICU patients – ones who are robustly well nourished upon admission, whose critical illness is not catabolic, and whose ICU stay is close to the average 3.8 days – the amount of protein and other nutrients they are provided is likely to be irrelevant. Any minimal benefits of instrumental specialized nutritional support could well be counterbalanced by its complications.

By contrast, catabolic critical illness dramatically increases obligatory N loss and decreases the efficiency of exogenous amino acid deposition into endogenous proteins, and it typically has a protracted time course. These observations strongly suggest that catabolic critical illness increases protein requirements.

The systemic inflammatory response releases a flood of amino acids from the muscles into the bloodstream, a process that transfers amino acids from the muscles to sites of injury and wound healing, and to the liver and other central tissues, which avidly take them up to synthesize acute-phase proteins involved in regulating the immunoinflammatory process. Except in settings of overwhelming sepsis or hepatic hypoperfusion, plasma amino acid concentrations are subnormal in unfed catabolic critically ill patients ^12, 13^. This is not a phenomenon of decreased muscle amino acid release (amino acid release from muscle is greatly increased) but rather is due to a dramatic increase in the rate of amino acid clearance from the circulation into the central tissues ^13– 15^. The overall physiological picture of catabolic critical illness that emerges is one in which the rapidly turning over central proteins (in the liver, splanchnic organs, bone marrow, and immunologically active tissues) take up and utilize amino acids as fast as the muscles release them. This is a portrayal of “acute central protein deficiency”, which suggests that sufficient exogenous amino acid provision could improve clinical outcomes by increasing central protein synthesis and, to some extent, mitigate the rapid muscle atrophy associated with catabolic critical illness. This picture also suggests that patients who are admitted to an ICU with pre-existing muscle atrophy (as from old age, inactivity, or protein-energy malnutrition) will be less able to mount a robust metabolic response to catabolic critical illness and hence are at higher risk of an adverse outcome unless provided a suitable amount of exogenous protein.

A second important feature of protein metabolism in catabolic critical illness is its extreme inefficiency. A large fraction of the amino acids that are released from the muscles are catabolized, either immediately or indirectly after entering the liver’s gluconeogenic pathway. Muscle protein loss vastly exceeds gains of protein mass elsewhere in the body, with the consequence that net body protein loss increases dramatically. Catabolic critically ill patients often excrete 15 g N or more in their urine every day in the absence of exogenous protein provision. This rate of body N loss implies a loss of 15 × 6.25 = 94 g muscle protein (and, since active protein tissues are approximately 20% protein and 80% water, approximately 0.47 kg of muscle mass) every day. This is extremely rapid muscle atrophy!

The third important feature of catabolic critical illness is very inefficient conservation of dietary amino acids. The same process that directs much of the flow of muscle-derived amino acids into catabolic pathways also directs exogenously supplied amino acids into these pathways. Consequently, very large doses of exogenous amino acids must be provided to achieve small net gains in body protein. The best that can often be accomplished is to minimize, rather than completely neutralize, the patient’s negative N balance.

Given our relatively sophisticated understanding of protein requirements in health and disease, and in light of the fact that critically ill patients don’t eat anything, the physiological case for prompt and generous protein provision is very strong. For more than four decades, there has been a wide consensus among clinical nutrition experts – based on a broad array of consistent animal and human metabolic data, supplemented by a small number of N balance studies that consistently indicate superior N balance when greater amounts of protein are provided, as well as observational data associating lower rates of protein provision with worse clinical outcomes – that catabolic critical illness greatly increases protein requirements ^13, 16– 23^.

## How much protein do critically ill patients need, and how much do they receive?

Notwithstanding the agreement among experts that catabolic critical illness increases protein requirements, the recommendations they offer for the specific amount of protein to provide to individual patients are vague and widely variable. The most recent guidelines of the Society of Critical Care Medicine and the American Society for Parenteral and Enteral Nutrition recommend providing most critically ill patients with 1.2 to 2.0 g protein/kg/day. They don’t explain how to select an individual patient’s dose within this wide range, other than to recommend more protein (as much as 2.5 g/kg) in certain specific situations ^22^. The European Society for Clinical Nutrition and Metabolism recommends 1.3 to 1.5 g/kg protein or amino acids/day for almost all critically ill patients ^24^. The evidence-based guideline group of the American Burn Association recommended 1.5 to 3.0 g/kg for severely burned patients ^25^. None of these recommendations appear to have been based on systematic reviews of the evidence. When a systematic review was formally carried out, it revealed that the recommendations in the guidelines were based on a small and unrepresentative subset of low-quality studies. Every study indicated that insufficient protein provision leads to serious and preventable body protein loss but, notwithstanding the claims made by some of their authors, none of them had sufficient quality or statistical power to rule in or out any specific level of protein or amino acid provision as maximally beneficial with regard to N balance. The main conclusion of the systematic review was that N balance improves with increasing protein provision to an upper limit of 2.5 g/kg/day ^13^. Finally, almost every study relied on metabolic rather than clinical outcome endpoints and hence cannot be regarded as definitive. The question of precisely how much protein to provide to catabolic critically ill patients can be answered only by carrying out well-designed, suitably powered clinical trials with hard clinical endpoints.

Despite the lack of high-quality clinical evidence regarding the specific amount of protein to provide to catabolic critically ill patients, the general recommendation that they promptly require generous amounts of protein is sound. The most frequently recommended specific protein target is 1.5 g/kg/day ^26^. At present, however, the patients in modern ICUs receive less than half this much protein – less even than a healthy person’s requirement – for at least the first two weeks of an ICU stay ^23, 27– 30^. This is an aberrant situation. How did it arise? Why does it persist?

## A brief history of ICU nutrition

Parenteral nutrition (PN) was developed in 1968 and widely adopted during the 1970’s ^31^. Aqueous mixtures of free crystalline amino acids, dextrose, micronutrients, and (when they became available) triglyceride emulsions were infused into the bloodstream through centrally located intravenous catheters. PN was advocated for most critically ill patients, although with the proviso that patients with an adequate muscle mass experiencing only mild catabolic stress had a lower priority than patients with protein-energy malnutrition experiencing intense catabolic stress ^32, 33^.

Metabolic studies in young, well-nourished adults with major traumatic injury or sepsis revealed dramatic increases of N loss and energy expenditure, and these findings were interpreted as implying that almost all critically ill patients should receive large infusions of amino acids and energy. Amino acid mixtures were combined with large amounts of dextrose for two reasons: firstly, to match presumed high rates of energy expenditure and, secondly, to stimulate insulin secretion as a strategy for increasing muscle protein anabolism ^31, 34– 36^. By the 1990’s, it became clear that energy expenditure is actually not increased very much in most critically ill patients and that overfeeding them with carbohydrate ^37– 39^ or fat ^40^ is harmful. During the same period, liquid nutrient formulas suitable for delivery into the gastrointestinal tract were developed and improved, and gastrointestinal feeding tube design and techniques became increasingly more sophisticated and effective. Head-to-head comparisons between enteral nutrition (EN) and PN indicated that EN was in general safer than PN (specifically with regard to the risk of infectious complications), so it became and remains the standard of care. PN came to be regarded as a treatment of last resort, with an emotional fervor fueled by editorials published in the mainstream medical literature with titles such as “death by parenteral nutrition” ^41^.

But there was an unintended consequence. EN turns out to have its own serious drawback: it provides too little, too late. Unlike with PN, which may be administered in a substantial dose soon after a central venous catheter is in place, EN requires insertion and placement verification of a plastic feeding tube followed by the slow, gradually increasing introduction of the nutrient solution into the stomach or intestines. The process is frequently interrupted by patient intolerance or for other reasons. The problem of inadequate protein delivery is compounded by the fact that, until very recently, the protein-calorie ratio of commercial EN products was appropriate for a normal person but seriously protein deficient for someone with catabolic critical illness. There’s clearly a problem. EN commonly fails to deliver adequate amounts of protein, while PN is fast and effective, but dangerous. What to do?

## The ICU as a nutrition lab

Academic medicine has long been hindered by a culture and educational system that fosters nutritional indifference and illiteracy ^42– 48^. No one disputes the evidence that micronutrient deficiency ^49^ and protein-energy malnutrition ^50– 53^ are highly prevalent in acute hospitals, but little has been done to address the situation. Decades of documentation haven’t persuaded hospital administrators to seriously modify their policies for nutrition provision (the changes necessary would be costly and probably require a different attitude about what hospitals are for) or triggered the high-quality prospective clinical trials necessary to demonstrate whether improved nutrition can actually improve clinical outcomes or shorten hospital stays. The ICU is an exception. The modern ICU has become a laboratory of nutritional research ^54^.

It’s worth recalling that the average minimum protein requirement of normal adults was determined from N balance studies ^11^. Nutrient requirement research on healthy, or at least ambulatory, people has to rely on biological indicators like N balance. By contrast, it is technically feasible to determine a protein target for acutely ill, hospitalized patients by carrying out clinical trials with hard clinical endpoints. Unlike with healthy people, patients can easily be monitored in an ICU, and their clinical status and health outcomes documented, and their disease trajectory is short enough to make clinical trials of different levels of protein (and other nutrients) feasible. The ICU enables a method of nutrition research that’s compatible with the ideal of evidence-based medicine (EBM): well-designed prospective randomized clinical trials (RCTs) with hard clinical endpoints.

## Growing pains

Experts in modern RCT methodology have become established in ICUs around the world; procedures for rigorous clinical data collection are in place. Large RCTs of critical illness nutritional interventions appear regularly in prestigious medical journals. Has this vigorous exercise in EBM resulted in new insights or improved clinical outcomes in the ICU? The answer appears to be a resounding no! What’s going on?

One of the questions the modern generation of RCT-skilled ICU nutrition investigators has tackled most vigorously is the one posed at the beginning of this article: what nutrition should be provided when a patient is admitted to an ICU, and how should it be provided? EN progresses too slowly. PN could easily make up EN’s shortfall, but there is unease about its increased risk of complications ^22, 31, 55^.

To address this question, several large RCTs were carried out that compared usual (inadequate) EN with the same EN supplemented by PN. There was no overall difference in clinical outcomes. Upon reviewing these trials, one author remarked that “Nutrition may be another treatment in the ICU where less is more” ^56^. A high-profile clinical practice review of these RCTs advised clinicians to continue the current practice of protein-calorie under-nutrition for at least the first week of a patient’s ICU stay, on the grounds that there is no evidence that doing otherwise improves clinical outcomes ^57^.

May we now conclude that underfeeding is the best nutrition early in an ICU stay? If so, how does this conclusion square with the decades of physiological and other evidence that favors early and generous protein provision? Has the rigorous finger of EBM pushed aside the senile, quavering stick of unreliable, “physiology-based medicine”?

Analysis of the nutrient composition of the PN regimens used in these trials leads to a different conclusion: *wrong nutrient*. The premise of these RCTs was that the only important macronutrient in critical illness is *calories*. Accordingly, their PN intervention arms infused large amounts of dextrose but very little protein substrate. Protein provision was grossly deficient in the control group of every trial (in accordance with current standard practice) but it was almost as severely deficient in the PN intervention groups ^58^. Except for one trial ^59^, PN-treated patients received less than half the most commonly recommended amount of protein ^26^. These RCTs showed that high-calorie, protein-deficient nutrition doesn’t improve clinical outcomes in the ICU ^60^. But they don’t support the recommendation that flowed from them to avoid early nutritional interventions *of any kind* because they ignored protein. (One of the PN-supplementation RCTs did indicate benefit from the intervention; it was the only one that provided a considerable amount of protein substrate, although still, in our view, inadequately ^58, 59, 61^.

It’s difficult to understand how clinical trial experts from several countries came to design large, ambitious RCTs that tested the biologically implausible hypothesis that infusing large amounts of calories and very little protein substrate into fat-replete or overweight, protein-deficient patients could improve their clinical outcomes ^58^. The authors of these trials either didn’t know about or ignored the vast amount of physiological and other evidence cited in this article, even though it convinced clinical nutrition societies in the United States ^22^ and Europe ^24^ to recommend generous protein provision in this setting.

One reason for the incompetent design of these RCTs appears to be a complacent ignorance of the details of nutritional physiology, presumably justified by the misconception that because physiological insight isn’t RCT “evidence”, it can be disregarded when designing clinical trials ^28, 62^. A second possibility is cognitive bias arising from a kind of stereotypical thinking that regards calories and nutrition as synonyms. A casual reading of the critical care literature turns up nearly countless examples of this conflation bias. For example, many modern RCTs were officially designated as “calories” trials ^59, 63– 65^ or referred only to calories in their abstracts ^66, 67^ but are now being interpreted as “nutrition” trials. When “calories” and “nutrition” mean the same thing, it’s easy to forget about the amount of protein in a nutritional regimen.

There’s a second way these RCTs went astray. It is a rule of medicine that therapy should flow from diagnosis. Only certain ICU patients would be strongly predicted to benefit from early high-protein provision. They are patients whose protein requirement is increased by catabolic critical illness and patients with pre-existing muscle atrophy when admitted to the ICU. None of these large RCTs obtained or analyzed their data in consideration of these easily obtainable and physiologically pertinent criteria.

A final situation in which critically ill patients are likely to benefit from generous protein provision is when calorie provision is deliberately limited. Energy deficiency is known to increase dietary protein requirements ^28, 68^. There is a considerable amount of clinical and observational evidence (but, as yet, no high-quality RCT evidence) that critically ill obese patients benefit from hypocaloric, high-protein, and even very high-protein nutrition ^22, 69^. Thanks to the crop of RCTs discussed in this article, we now have strong RCT evidence that most critically ill patients, not just the obese ones, are unharmed and may be best served by hypocaloric nutrition during the catabolic phase of their disease. Physiological logic dictates that most patients who are hypocalorically fed, not just obese ones, would likely benefit from generous protein provision.

## Growing up

The ICU as a nutrition lab is a valid, exciting venture in clinical nutrition research. It’s normal to experience growing pains when first applying a model – the RCT – that was developed to evaluate single-drug treatments for well-defined diseases to the complex and heterogeneous landscape of nutrition and “critical illness”. Modern RCT-skilled clinical investigators are to be congratulated for their dedication to the work of rigorous RCT design and execution, but they require a more sophisticated comprehension of, and respect for, nutritional physiology. All therapeutic trials – but especially nutritional ones, given their complexity – should be founded on physiologically sound premises ^28, 62^. Providing calorie-rich, protein-deficient nutrition to protein-deprived, fat-rich ICU patients fails the fundamental test of physiological rationality.

Modern clinical trial experts will have done good service if their recent RCTs spur the clinical nutrition community to seriously address the question of appropriate protein provision in critical illness in general and catabolic critical illness in particular. The current controversy clearly shows the desperate need for nutritionally literate, high-quality RCTs to confirm or refute the effectiveness of specific high-protein nutritional regimens for critically ill patients whose metabolic profile predicts they are likely to benefit ^13, 28, 70– 72^.

Two relevant RCTs were recently published ^73, 74^. One of them ^73^ compared hypocaloric nutrition that provided either 92 or 45 g protein/day. Although the trial lacked statistical power, delivered an inadequate amount of protein, and enrolled patients with non-catabolic critical illness, biomarker and other indicators nevertheless suggested better outcomes for the patients who were provided 92 g protein/day. The second RCT compared standard EN (0.8 g protein/kg/day) with standard EN plus an intravenous mixed amino acid supplement which, when combined with EN, provided approximately 1.6 g protein/kg/day to critically ill patients with renal dysfunction ^74^ (the published article overstates the amount of protein administered in the EN plus PN group, failing to appreciate that the molecular weight of the hydrated amino acids in PN mixtures is 18 mass units greater than that of peptide-bound amino acids; the amino acids in PN contain 17% less protein substrate than a similar mass of formed protein ^75^). The aim of the RCT was to determine whether increased amino acid provision would foster a better preservation of renal function; it did not. This well-designed and -implemented RCT focused on renal physiological effects and its endpoint was a biological marker; it was not powered to detect differences in patient-centered clinical outcomes. From the protein-requirement perspective, it can be faulted for failing to define “critical illness” other than being in an ICU and for failing to determine and consider the muscle mass and protein-catabolic intensity of the patients enrolled in it. While imperfect, these RCTs represent a salutary beginning.

Until new protein-enriched EN products have clearly been shown to be capable of promptly delivering suitably generous amounts of protein to critically ill patients without simultaneously delivering excess calories ^20, 28, 76^, optimum protein dose-finding studies will have to rely on PN to make up EN shortfalls. It is increasingly likely, but still to be confirmed, that when PN is used prudently and without calorie overfeeding, it is as safe as EN ^22, 28, 31, 55, 65, 77, 78^ and, for many patients, more effective at delivering sufficiently generous amounts of amino acids.

Urinary N excretion indicates the severity of a patient’s protein-catabolic state ^16, 20, 79^ and thus identifies patients whose protein requirement is increased. Despite its technical simplicity, urinary N excretion is rarely measured in modern ICUs. In current practice, the severity of critical illness is quantified using a variety of scoring systems ^80, 81^ with the assumption that they predict protein-catabolic intensity, but they don’t ^16, 20, 82^. These scores were developed and validated to predict death, not N loss. A newly developing scoring technique, called NUTRIC, could prove useful in predicting which ICU patients (whether catabolically critically ill or not) are most likely to benefit from intensified nutrition support ^83^. The NUTRIC score doesn’t include N excretion, a practical advantage because N excretion is rarely measured in ICUs but a drawback because of the obvious nutritional relevance of identifying catabolic critically ill patients.

It is easy to evaluate a patient’s muscle mass at the bedside by simple physical examination ^84, 85^. Serum creatinine concentration and urinary creatinine excretion predict muscle mass ^86– 88^, although their interpretation can be confounded by concurrent renal injury. Bedside muscle imaging offers considerable promise ^89^. None of these determinations are commonly carried out in current ICU practice.

## Conclusions

Modern ICU patients tend to be fat rich and protein poor, the latter due to generalized muscle atrophy that preceded their current critical illness. Incomprehension of the nutritional implications of being fat rich and protein poor, coupled with an insistence on using protein-deficient EN as the sole route of nutrient delivery, has created a state of affairs in which critically ill patients are delivered calories they don’t need and deprived of the protein they may well need. Except for frankly obese patients, energy targets are routinely set too high and protein targets ignored. Energy expenditure is routinely measured or estimated in order to set calorie targets, but N loss is rarely determined, even though this simple measurement identifies the catabolic critically ill patients who are most likely to benefit from generous protein provision. Nutritional assessment too often focuses on adipose tissue mass while ignoring the muscle atrophy that creates a much more serious nutritional risk.

The modern ICU has become a laboratory of high-quality nutrition research. RCT-skilled ICU nutrition experts require a better comprehension of and respect for nutritional physiology. Providing calorie-rich, protein-deficient supplemental nutrition to protein-starved, fat-rich ICU patients fails the fundamental test of physiological rationality. The current controversy over optimum nutrition in the ICU highlights the need for nutritionally literate, high-quality RCTs to confirm or refute the effectiveness of specific high-protein nutritional regimens for catabolic critically ill patients.
